# Monitoring Of High-Dose Methotrexate (Mtx)-Related Toxicity and Mtx Levels in Children with Acute Lymphoblastic Leukemia: A Pilot-Study in Indonesia

**DOI:** 10.31557/APJCP.2021.22.7.2025

**Published:** 2021-07

**Authors:** Nur Melani Sari, Lulu E. Rakhmilla, Muhammad Hasan Bashari, Zulfan Zazuli, Nur Suryawan, Susi Susanah, Lelani Reniarti, Harry Raspati, Eddy Supriadi, Gertjan J L Kaspers, Ponpon Idjradinata

**Affiliations:** 1 *Hematology Oncology Division, Department of Child Health Faculty of Medicine Universitas Padjadjaran/Dr. Hasan Sadikin General Hospital, Bandung, Indonesia. *; 2 *Department of Public Health, Division of Epidemiology and Biostatistics, Faculty of Medicine Universitas Padjadjaran, Bandung, Indonesia. *; 3 *Department of Biomedical Sciences, Division of Pharmacology and Therapy, Faculty of Medicine, Universitas Padjadjaran, Bandung, Indonesia. *; 4 *Department of Pharmacology-Clinical Pharmacy, School of Pharmacy, Bandung Institute Technology, Bandung, Indonesia. *; 5 *Amsterdam UMC, location Academic Medical Center, Amsterdam, Netherlands. *; 6 *Pediatric Hematology Oncology Division, Department of Pediatrics, Dr Sardjito Hospital-Faculty of Medicine Public Health and Nursing Universitas, Gajah Mada, Yogyakarta, Indonesia. *; 7 *Princess Máxima Center for pediatric oncology, Utrecht, Netherlands. *; 8 *Emma Children’s Hospital, Amsterdam UMC, Vrije Universiteit Amsterdam, Pediatric Oncology, Netherlands, Netherlands.*

**Keywords:** Acute lymphoblastic leukemia, high dose methotrexate, toxicity, 2013 ALL Indonesian protocol

## Abstract

The administration of high-dose methotrexate (HD-MTX) requires an accurate monitoring of blood MTX levels to determine the regimen of leucovorin rescue and urine alkalinization to prevent toxicity. However, it is technically and logistically challenging to screen patients routinely in limited-resource settings. This study aimed to evaluate blood MTX levels at 24- and 48-hours from start of infusion in relation to clinical toxicity in childhood ALL.

**Methods:**

A prospective cohort study was conducted on 32 consecutive children with acute lymphoblastic leukemia (ALL) who had received at least one cycle of 1 g/m^2^ HD-MTX intravenous infusion as a part of consolidation treatment based on the 2013 Indonesian ALL Protocol. In total, 68 cycles were evaluated. Serum MTX concentrations were measured using enzyme immunoassay. MTX toxicity was categorized using common toxicity criteria (CTCAE) 3.0 version. The association between MTX level and clinical toxicity was assessed by non-parametric analysis.

**Results:**

The 24-hours MTX level was median 29.8 ng/mL (0.065 µmol/L) (IQR 8.1–390.6) with a modest decrease in 48-hours MTX serum level in all cycles (median 28.3 ng/mL and 0.062 µmol/L; IQR 0.35–28.7; p<0.05). The two most common toxicities were hepatotoxicity (32.2%) and neutropenia (30.9%). Nephrotoxicity and febrile neutropenia occurred in 8.8% and 5.8% of patients, respectively, with low percentage of mucositis (4.3%) and thrombocytopenia (5.6%) recorded. No statistically significant association was found between MTX levels and clinical toxicity, except for liver toxicity.

**Conclusion:**

Serum MTX levels at 24-hours and 48-hours are low, followed by only 4.4% grade III/IV hepatotoxicity and 26,4% grade III/IV neutropenia. There is no significant association between the clinical toxicity and MTX levels at the two points of measurement. An attempt to increase the MTX dose and/or to introduce a loading dose should be considered in subsequent ALL protocol as supported by further pharmacokinetic MTX studies in the Indonesian population.

## Introduction

Until currently, HD-MTX therapy with repeated intrathecal injections has been generally accepted as an elective regimen for preventing central nervous system involvement and acts as a cornerstone of treatment in children with ALL (Mantadakis et al., 2005). However, high-dose methotrexate (HD-MTX), defined as a dose higher than 500 mg/m^2^, is still a challenging regimen for pediatric oncology patients in some developing countries (Treon and Chabner, 1996; Moe, 2000; Zangooei et al., 2013; Ferdousi et al., 2017). The efficacy and toxicity of HD-MTX in an acute lymphoblastic leukemia (ALL) treatment protocol are two sides of a coin that should be considered for successful treatment (Mantadakis et al., 2005; Howard et al., 2016). HD-MTX needs close monitoring for various parameters, which creates a challenge for pediatric oncologists in developing countries due to the lack of resources to routinely screen patients (Jo¨nsson et al., 2011; Foster et al., 2017; Gauri Kapoor et al., 2012). This has made many pediatric oncologist reluctant to use MTX or modify its dosage (Gauri Kapoor et al., 2012).

Indonesia faces various medical and non-medical challenges in treating children with leukemia. High dropout rate, interrupted treatment, high premature deaths, and inadequate hospital logistics are some of the contributors of the dismal outcome of pediatric leukemia in Indonesia when compared to the outcome of the developed countries (Rivera and Ribeiro, 2014; Gupta et al., 2015; Sari et al., 2015; Handayani et al., 2016). Clinicians are hesitant to increase the dose of MTX because there is a high risk of infection and malnutrition as comorbidities. Moreover, there are very limited efforts to monitor drug levels in Indonesia. Studies have indicated that the number of cycles and the MTX dosage administered is lower (< 1g/m^2^) in the protocols of developing countries, including in Indonesia, which may lead to less effective treatment (Ferdousi et al., 2017; Agnes et al., 2 018; Tiwari et al., 2018).

The dose for HD-MTX in Indonesian ALL Protocol has been modified from 500 mg/m^2^ in 2006 to 1,000 mg/m^2^ continuous infusion without loading dose, in 2013 (Wijayanti and Supriyadi, 2017; Yakin et al., 2017).

Unfortunately, until the fifth year of implementation, only a few studies were carried out to evaluate the protocol outcome, with most of them mainly focus on HD-MTX dose provision, without studying toxicity or efficacy. Previous studies showed that the three-year survival rate of ALL treatment administered under the 2006 and 2013 Indonesian protocols is still ranging between 22–50%, with no difference found between the inputs and outcomes of the two protocols (Wijayanti and Supriyadi, 2017; Yakin et al., 2017). The paucity of data on the administration of HD-MTX has prompted the need to do a study to capture the primary data in order to improve the future ALL protocol, especially regarding the administration of high-dose methotrexate, as well as to highlight the importance of MTX serum level measurement. Hence, this study aimed to evaluate the HD-MTX levels in blood at 24- and 48-hours and identify the clinical HD-MTX related toxicity in children with ALL.

## Materials and Methods

This pilot study was a prospective observational cohort study aimed to evaluate the clinical toxicity of HD-MTX in children with ALL treated under the 2013 Indonesian ALL Protocol in the consolidation phase. This study was conducted at Dr. Hasan Sadikin General Hospital Bandung, Indonesia with one-year observation starting from August 2018. The inclusion criteria used were children with ALL who were treated with HD-MTX during the consolidation phase. Informed consent was obtained from the parents/guardians. Demographic, clinical, and clinical outcome data were then collected. The clinical toxicity of adverse event (AE) was assessed according to the National Cancer Institute common toxicity criteria version 3.0 (CTCAE) that include the incidence of febrile neutropenia (grade 3-4), reduced neutrophil count, reduced platelet count, nephrotoxicity, and increment of liver function (National Cancer Institute, 2003).

All parameters were measured before the following cycle (7–21 days post-HD-MTX administration) based on parental report, physical examination, and laboratory results. Blood samples were collected at 24 and 48-hours after the starting of MTX infusion, followed by centrifugation. Samples were then frozen at -80°C and stored for a maximum of six months for MTX assay. The MTX level was measured using the “Qayee-Bio” Human Methotrexate (MTX) ELISA Kit with a sensitivity range of 9.3–300 ng/mL. This kit is a homogenous enzyme immunoassay intended for quantitative analysis of human serum or plasma MTX. The measurement was performed according to the procedure from the manufacturer, which is also the same method used in a previous study in Indonesia (Utomo et al., 2017). The concentration gained was then converted into µmol/L by multiplying it with 0.0022 for comparison with other studies. Nephrotoxic medications, such as NSAIDs, and co-trimoxazole, were avoided during the HD-MTX infusion until the drug was already cleared. 

**Figure 1:**
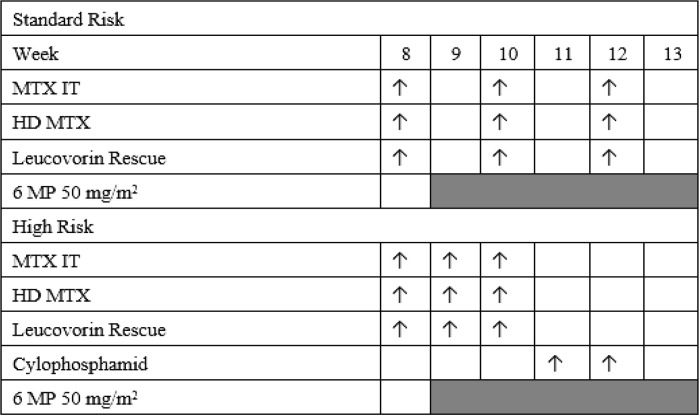
Consolidation Phase in 2013 ALL Indonesian Protocol, (Hematology Oncology Working Group Indonesian Pediatric Society, 2013)

All patients in this study received 1,000 mg/m^2^ MTX therapy based on the 2013 Indonesian ALL protocol (Figure 1). Patients received intrathecal (IT) MTX therapy during or after the dose was completed with the dosage adjusted to the patient’s age. Patients also received hyperhydration therapy and pre-methotrexate urine alkalinization (pH >6.5). High-dose methotrexate was dissolved into saline infusion based on 2–3 L/m2 hydration and administered for 24 hours intravenously without a loading dose. After the administration of HD-MTX, patients also received hydration followed by eight doses of leucovorin rescue (15 mg/m^2^/dose) every 6 hours at 42 hours after starting the procedure by empirical administration since it was not possible to measure serum MTX concentrations in all oncological units in the country.

*Data Analysis*

Continuous variables were summarized as median, range, and interquartile while categorical variables were presented as numbers and percentages. MTX serum levels at 24 and 48 hours were consolidated with each adverse event. Variables were compared using non-parametric tests such as chi-square, bivariate Mann-Whitney, and Kruskal-Wallis tests. In this analysis, one observation for each patient was considered. The findings were considered statistically significant at p-values of <0.05.

## Results

The study group comprised of 32 consecutive patients who had received at least one cycle of HD-MTX and who received a total of 68 cycles of HD-MTX during the study period. Of these, not all patients receive 3 cycles of MTX therapy. During the inclusion period, some patients started on a second or third cycle. Some also only underwent one cycle due to treatment abandonment. Of the 32 ALL patients included in this study, there were 20 females and 12 males. Their median age at diagnosis was five years (range, 1-16 years). The most common HD-MTX induced toxicity were hepatotoxicity (32.2%) and neutropenia (30.7%), while the least common was mucositis (4.3%). Most of the hepatotoxicity occurred in grade I (20.5%), while neutropenia was found in all grades and the most common was grade 3 (14,7%). Mucositis occurred more at grade 1 rather than grade 4 (Tables 1 and 2).

No significant difference was found between the median MTX serum levels at 24- (p value = 0.588) and 48-hours (p value = 0.474) after the start of infusion for each cycle (Table 3). However, there was a decrease in the MTX serum level between 24 and 48 hours in all cycles analyzed (p value = 0.005). Serum MTX levels varied between patients and within individual patients.

Generally, toxicity was mild and most patients were discharged within 72 hours without complications. The frequency of various toxicities observed with HD-MTX are shown in Table 3. No lethal toxicity was observed. Transient elevations of transaminases (32.2%) and neutropenia (mild to moderate) were the most frequent toxicities. The manifestations of toxicity were not associated with the MTX level at 24- and 48-hours, except for liver toxicity.

**Table 1:** Clinical and Initial Laboratory Manifestation

Characteristic	n= 32	(%)
Sex		
Male	12	37.5
Female	20	62.5
Age, years {range (median)}	1–16 (5)	
Risk Stratification		
Standard Risk	14	43.7
High Risk	18	56.3
WBC at Presentation		
< 50,000	28	87.5
≥ 50,000	4	12.5
FAB Classification		
L1	2	6.2
L2	17	53.1
L3	0	
Undetermined	13	40.6
Nutritional Status		
Malnutrition	7	21.8
Normal	25	78.1
Outcome		
Death and Relapse during Treatment	11	34.4
On Treatment	21	65.6

**Table 2:** Clinical Toxicity Characteristics

Clinical Toxicity	n=68 events	(%)
Mucositis		
Grade 1	2	2.9
Grade 2	0	0
Grade 3	0	0
Grade 4	1	1.4
Neutropenia		
Grade 1	1	1.4
Grade 2	2	2.9
Grade 3	10	14.7
Grade 4	8	11.7
Febrile Neutropenia		
Grade 3	3	4.4
Grade 4	1	1.4
Thrombocytopenia		
Grade 1	1	1.4
Grade 2	1	1.4
Grade 3	1	1.4
Grade 4	1	1.4
Nephrotoxicity		
Grade 1	5	7.3
Grade 2	0	0
Grade 3	1	1.4
Grade 4	0	0
Hepatotoxicity		
Grade 1	14	20.5
Grade 2	5	7.3
Grade 3	3	4.4
Grade 4	0	0

Notes: *Nephrotoxocity*, A disorder characterized by the acute loss of renal function and is traditionally classified as pre-renal (low blood flow into kidney), renal (kidney damage) and postrenal causes (ureteral or bladder outflow obstruction). Grade 1: Creatinine level increase of >0.3 mg/dL; creatinine 1.5–2.0 x above baseline. Grade 2: Creatinine 2–3 x above baseline. Grade 3: Creatinine >3 x baseline or >4.0 mg/dL. Life-threatening consequences; dialysis indicated. Hepatotoxicity: A disorder characterized by acute loss of liver function. Grade 1: >ULN – 3.0 x ULN; Grade 2: >3.0 – 5.0 x ULN; Grade 3: >5.0 – 20 ULN

**Table 3:** Median Serum MTX Levels and IQR at 24- and 48-hours by Treatment Cycle

Time After Start of Infusion	24 hours (Median) ng/mL	p#	48 hours (Median) ng/mL	p#	p*
Cycle 1 (n: 27/32)	29.9 (IQR 8.1–327.5)	0.588	27.4 (IQR 0.35–228)	0.474	
Cycle 2 (n: 20/32)	29.2 (IQR 16.0–334.4)		27.8 (IQR 3.2–280.6)		
Cycle 3 (n: 21/32)	30 (IQR 14.6–390.6)		32.9 (IQR 14–252.3)		
All cycles (n: 68) *	29.8 (IQR 8.1–390)		28.3 (IQR 0.35–280.7)		0.005

*, Analysis by cycle at 24- and 48-hours; #, Analysis by each cycle

**Table 4:** Association between Clinical Toxicity and MTX Levels (Median and IQR)

Cycles (n: 68)	24-hours MTX level (ng/mL)	p	48-hours MTX level (ng/mL)	P
Mucositis				
With Mucositis (n: 3)	38.3 (IQR 27.2–51.4)	0.610	32.8 (IQR 0.35–280.6)	0.475
Without Mucositis (n: 65)	29.8 (IQR8.2–390.6)		27.8 (IQR 28.0­–76.5)	
Neutropenia				
With Neutropenia (n: 47)	29.9 (IQR 14.6–390.6)	0.392	27.2 (IQR 13.95–252.3)	0.637
Without Neutropenia (n: 21)	29.7 (IQR 0.35–280.6)		29.8 (IQR 0.35–280.6)	
Febrile Neutropenia				
With FN (n: 4)	32.8 (IQR 14–252.3)	0.831	32.2 (IQR 24–42.2)	0.85
Without FN (n: 64)	29.7(IQR 8.1–344.4)		28.3 IQR (0.4–280.6)	
Thrombocytopenia				
With Thrombocytopenia (n: 4)	35.4 (IQR 16.7–51.4)	0.890	36.3 (IQR17.3–76.5)	0.734
Without Thrombocytopenia (n: 64)	29.8 (IQR 8.1–390.6)		27.9 (IQR 0.4–280.6)	
Hepatotoxicity				
With Liver Toxicity (n: 22)	36.1 (IQR 11–390.6)	0.018	32.8 (IQR 17.3–76.5)	0.001
Without Liver Toxicity (n: 46)	28.3 (IQR 8.1–37)		21.1 (IQR 0.4–260.8)	
Nephrotoxicity				
With Nephrotoxicity (n: 6)	29.9 (8.2–37)	0.44	28.3 (IQR 0.35–40.2)	0.63
Without Nephrotoxicity (n: 62)	29.5 (11–390.6)		28.3 (IQR 3.2–280.6)	

## Discussion

To the best of our knowledge, this was the first study to measure the MTX levels and its correlation with clinical toxicity in 2013 ALL Indonesian Protocol. In this study, the median MTX level at 24 hours was 29.8 ng/mL (0.065 µmol/L), which then decreased to 28.3 ng/mL (0.062 µmol/L) at 48-hours. Although this decrease was statistically significant, it did not have significant impact clinically. In comparison to other studies that also evaluated ALL treatment protocols using the same dosage, these median MTX levels are lower (Joannon et al., 2004; Whitehead et al., 2005). In an evaluation on PANDA protocol, it was observed that the mean MTX level at 24 hours was 12.17 µmol/L, which then decreased to 0.37+0.20 µmol/L at 48 hours (Joannon et al., 2004). Meanwhile, an evaluation of POG Protocol 9005 presented a median plasma methotrexate concentration of 10.4 µmol/L at 24 hours (Mantadakis et al., 2005; Salzer et al., 2012). Another study from our country on the same protocol also found the same low MTX level trend at 24 hours and 48 hours of 10.73–175 ng/mL and 10.06–243.44 ng/mL, respectively (Utomo et al., 2017). 

The methotrexate pharmacokinetic study using the same dose was initially demonstrated in Evans et al., (1986) where they used 1 gram of MTX with an initial loading dose followed by continuous infusion up to 24 hours. In this study, 55% of the study population experienced fast clearance with a consistently low MTX level of below 20 µmol/L. This is important to note because a concentration below 16 µmol/L at 24 hours has been associated with the risk for relapse (Evans et al., 1986) and because an adequate extracellular concentration has been reported to be necessary for substantial passive uptake of methotrexate by human lymphoblastoid cells to prevent drug resistance (Evans et al., 1984). It has been reported in another study that the steady-state concentration of MTX at 1 g/m^2^ was 21 µmol/L (BorsiI and Moe, 1987). However, Wang et al., (1976), using 500 mg/m^2^ MTX dose, has reported that providing 1/3 bolus IV as a loading dose could increase the steady-state concentration of MTX from 0 to approximately 30.5 µmol/L in 30 minutes (Wang et al., 1976; BorsiI and Moe, 1987). This highlights the importance of providing loading dose to achieve the necessary steady-state concentration since providing continuous HD-MTX without bolus IV could prolonged the time needed to achieve the necessary concentration or even lower it (Wadhwa and Cascella, 2020).

Various studies have shown that the disposition of methotrexate in the body has a high inter-individual variability that may be influenced by, among others, the genetic polymorphisms for influx-efflux transporters, drug-metabolizing enzymes, and drug targets which affect the pharmacokinetics and pharmacodynamics of MTX (Ahmed et al., 2016). Studies have also assumed that the kinetic variability after a high dose of methotrexate administration may also be due to the rapid elimination that will decrease peak plasma concentration and duration of the effect (Evans et al., 1984; William E. Evans et al., 1998). ATP-binding cassette (ABC) transporters, i.e. ABCC2, ABCC3, and ABCG2, are the transmembrane proteins involved in rapid elimination of MTX and 7OH-MTX after intravenous administration and can, to a large extent, compensate for each other’s absence. However, the risk for highly increased toxicity due to the dysfunction of ABCC2, ABCC3, or ABCG2 alone is limited, as shown in a study using experimental mice (Vlaming et al., 2009). A study on Malaysian population has identified the ABCC2 genotype to be significantly associated with increased serum MTX levels at 48-hours after treatment and that MTX toxicity was seen in 5 g/m^2^ dose (Razali et al., 2020). However, this needs to be confirmed for Indonesian population through a pharmacokinetic study using a smaller MTX dose.

Delayed clearance has long been known to increase the risk for myelotoxicity (>0.1 µmol/L in 48-hours). In our study, only 1 patient (3 events) experienced delayed clearance with mild toxicity (grade 1 neutropenia, and hepatotoxicity). Although a significant decrease in the MTX level at 48-hours was seen, it was not as low as the levels seen in previous MTX pharmacokinetic trend studies which were much lower that our result (Evans et al., 1984; Evans et al., 1986; BorsiI and Moe, 1987). Intrathecal (IT) MTX after the end of 24-hours was shown to influence the pharmacokinetic trend. The procedure in the protocol resulted in increasing plasma level that reached the peak 3 to 12 hours after injection, followed by decreasing level with a half-life of 5.5 to 24 hours. This is due to the interaction of convective transport and diffusion between cerebrospinal liquids and brain extracellular fluid. Another study found a serum MTX concentration that ranges between undetectable up to 0.11 µM/L for the same IT dose at 15–24 hours in seven control patients with normal renal function. They found the concentration to be undetectable at 48 hours in all controls (Borsi et al., 1990; Gregory et al., 1991).

Leucovorin rescue dosage should be one of the consideration when determining HD-MTX efficacy since cytotoxicity is determined by three factors: drug concentration, duration of exposure, and leucovorin rescue (Chabner and Longo, 2011). An excessive leucovorin rescue dose may reduce toxicity but at the same time may decrease the desired anti-tumoral effect of MTX by influencing the folic acid concentration. The timing and dose of leucovorin rescue are essential, with both too low and too high doses of leucovorin are detrimental for the cure. A low dose of leucovorin leads to the appearance of MTX side effects and, more importantly, could increase mortality. On the contrary, high-dose leucovorin neutralizes the anti-tumoral effects of MTX and thus allows the malignant cells to relapse in CNS or elsewhere. In the Indonesian protocol, the dosage for leucovorin is eight doses (15 mg/m^2^) in a 6-hour interval. Considering the low MTX level at 24-hours and the small portion of delayed MTX clearance, this leucovorin dosage is excessive (Skarby et al., 2006). It is suggested to use only 2 doses of leucovorin with the current MTX dose, which might be beneficial to achieve better therapeutic results with high-dose methotrexate (Borsi et al., 1990; Cohen, 2013).

The two most common HD-MTX related toxicities identified in this study were neutropenia and liver dysfunction, which are consistent with those in previous studies using the same HD-MTX dosage (Khan et al., 2012). Even with a higher dose (3 g/m^2^), several other studies also did not find any correlation between serum MTX and clinical toxicity, especially the renal toxicity (Gauri Kapoor et al., 2012; Tsurusawa et al., 2015; O¨zdemir et al., 2016). Hepatotoxicity is also observed in another Indonesian study (25%) after the administration of high-dose MTX (Maharanto, 2016).

Previous studies have reported that the use of HD-MTX with an increased dose of 6-MP (mercaptopurine) may also increase hematotoxicity and hepatoxicity (Niekerk et al., 2008; Levinsen et al., 2015). Another study also demonstrated a possible pharmacodynamic interaction between MTX and 6-MP. It is suggested that MTX may increase the bioavailability of 6-MP and also, through inhibition of de novo purine synthesis, enhance the availability of 6-thioguanine and its incorporation into the DNA (Schmiegelow and Bretton-Meyer, 2001). The dosage of MTX usage in Indonesia Protocol is 50 mg/m^2^.

This study possesses several limitations. It is difficult to compare the results with results from other studies because, unlike the previously published studies, a loading dose was not a part of the protocol used in this study. Moreover, the measurement tools were also different, which is further complicated by a high number of patients who dropped out during the observation. During the period of this study, no pharmacokinetic laboratory was available to facilitate MTX assay in all of Indonesia, and the ELISA kit was the only accessible measurement tool available. There was also no pharmacokinetic study that described the effect of genetic variations in Indonesian population and there was only one publication on MTX pharmacogenetic study in Indonesian population (Giovannetti et al., 2008). Indonesian children have a higher proportion of 3R allele which makes them less sensitive to MTX. 

Thus, this study shows relatively low levels of MTX in children with ALL after continuous infusions of 1,000 mg/m^2^, without a loading dose. Modifications must be considered aiming at a higher efficacy of this important treatment element in future Indonesian protocol for ALL. Introducing a loading dose is one option, and higher dose as well. Of course, such modifications should be carefully monitored for MTX levels, clinical toxicity, and efficacy.

In conclusion, The level of 24-hour MTX in our study is low with generally mild MTX-induced toxicity. There is no significant association identified between the clinical toxicity and the MTX levels at 24 hours and 48 hours. Only 3/68 cycles with delayed clearance but without severe clinical toxicity has been observed. The use of a loading dose and a higher MTX dose should be considered to be incorporated into the Indonesian ALL protocol with the support from further pharmacokinetic MTX studies in Indonesian population.

## Author Contribution Statement

NMS, NS, and PT designed and reviewed the results. LER and MHB develop research method and data analysis. NMS, ZZ, SS, LR ES and GJLK wrote and revised the manuscript. All authors read and approved the final version of the manuscript.
